# The Most Comfortable Posture at First Postnatal Day in Women With Episiotomy for Breastfeeding and Routine Activities

**DOI:** 10.7759/cureus.13432

**Published:** 2021-02-18

**Authors:** Debashree Dutta, Sarita Singh, Priyanka Naik

**Affiliations:** 1 Obstetrics and Gynaecology, Vardhman Mahavir Medical College and Safdarjung Hospital, New Delhi, IND

**Keywords:** episiotomy, perineal pain, vas

## Abstract

Background: Episiotomy is a common procedure in vaginal deliveries. It was thought that episiotomy reduces the risk of severe perineal injuries, but now in various studies it has been observed that it is associated with increased perineal pain, sexual problems, and incontinence in the postpartum period.

Purpose: This study was carried out to assess pain score on first postnatal day in women with episiotomy during breastfeeding and daily activities.

Method: The consecutive patients who underwent normal vaginal delivery with episiotomy from November 1, 2020 to December 1, 2020 in the Department of Obstetrics and Gynecology at Safdarjung Hospital were evaluated. The patients with vulval edema; vulval hematoma; instrumental delivery; and cervical, vaginal, and perineal tear were excluded from the study. The episiotomy pain during routine activities and breastfeeding was assessed at first postnatal day by Visual Analogue Scale (VAS) pain score. The analysis of variance (ANOVA) was used to compare the VAS score in different positions for breastfeeding and routine activities. The most comfortable posture was with lowest VAS pain score.

Results and conclusion: Overall 73 patients were enrolled in the study, mean age was 23.48 ± 2.49 years, 47/73 (64.38%) were primigravida, and mean birth weight of neonate was 2.79 ± 0.43 kg. The mean VAS pain score during breastfeeding in crossed-leg position was 74.82 ± 28.33, and in left lateral position, the score was 14.51 ± 11.65 (p [U1] = 0.0001). The mean VAS pain score during resting position in sitting with leg down was 56.52 ± 21.23, and in left lateral position, the score was 21.25 ± 13.95 (p= 0.0001). The VAS score was increased as the birth weight (p= 0.302), and the duration of second stage of labor was increased (p = 0.047). The severe pain was in 5/73 patients using Western toilet and in 46/73 Indian toilet (p≤ 0.0001).

Conclusion: The left lateral position for breastfeeding and resting supine are least painful positions. The Western-style toilets are more comfortable than Indian-style toilets.

## Introduction

Episiotomy is a common procedure in vaginal deliveries and is defined as the incision on perineum in order to provide enough space for delivery and thereby eliminating the risk of severe perineal injuries [1-3].

The women experiencing episiotomy varies from 10% to 95% in studies. In our hospital, a tertiary care referral center, about 1800-1900 births occur monthly, out of which nearly two-third of these births are vaginal births, and episiotomy is performed on the majority of primigravida women.

In systematic reviews it has been reported that episiotomy is not as beneficial as expected, and it does not decrease the risk of perineal trauma and should not be routinely performed. Moreover episiotomy is associated with increased perineal pain, sexual problems, and incontinence in the postpartum period [1-3]. The perineal pain due to episiotomy interferes with daily activities and care of the newborn baby. The severe perineal pain can occur in up to 90% of postpartum women; it can persist upto six weeks after delivery in nearly 25% of women. The methods used for relieving the perineal pain are oral analgesics, nonsteroidal anti-inflammatory drugs (NSAIDs), and ice bags [4].

The relation of posture with perineal pain has not been studied, and the present study was carried out not only to find the association of perineal pain with postures and activities but also to find whether a mother’s most comfortable position is compatible with breastfeeding and kangaroo care.

## Materials and methods

This was a cross-sectional study for a period of one month, and patients who were admitted in the Obstetrics and Gynecology department of Safdarjung Hospital, Delhi, India, were evaluated. The patients who underwent normal vaginal delivery with right medio-lateral episiotomy within 4-24 hours after the delivery were included in the study. The patients with vulval edema; vulval hematoma; instrumental delivery; and cervical, vaginal, and perineal tear were excluded from the study.

The written informed consent was taken from all study participants, and ethical clearance from the institute ethics committee was obtained prior to starting the study. Data were collected using predesigned questionnaire-based pro forma having demographic and obstetrics data. The Visual Analogue Scale (VAS) - a standard score for measuring pain intensity in clinical situations - was used for measuring the post episiotomy perineal pain. The VAS measures pain on a scale from zero to 100 with zero representing “no pain,” 10-30 “mild pain,” 40-60 “moderate pain,” and 70-100 as “severe pain.” The VAS pain score during breastfeeding was measured in six different positions used. The postures used during breastfeeding were legs crossed, lying supine position, left lateral, right lateral, sitting with leg down, and sitting with leg straight on bed. The VAS pain score during routine activities was also done in these positions, i.e., lying in supine position, left lateral, right lateral, sitting with leg down, sitting with leg straight, and walking. The VAS score during the use of Indian or Western toilet was also measured.

The repeated measure one-way analysis of variance (ANOVA) was used to compare pain score during different breastfeeding positions and other routine activities. The most comfortable position for breastfeeding was identified by least VAS score. The other activities were graded as per VAS score starting with the lowest pain score. The VAS score during the use of Western toilet and Indian toilet was also compared. p value ≤ 0.05 was taken as statistically significant. The data analysis was done using Statistical Package for Social Sciences (SPSS) version 21.0 (IBM Inc., Armonk, USA).

## Results

On the basis of pilot study, taking a population size of 219 and mean difference in pain as 5 with standard deviation of 10 between different postures, the calculated sample size with 90% power of study and 5% level of significance was 73 patients.

**Baseline characteristics**

The mean age of patients was 23.48 ± 2.49 years, and 64.38% were primigravidas. The mean period of gestation at the delivery was 38.28 ± 1.88 weeks, and the mean birth weight was 2.79 ± 0.43 kg (Table 1).

**Table 1:** Baseline Demographic, Obstetrics, and Neonatal Characteristics of Participants

Variables	Results
Age (years)	23.48 ± 2.49 years
Primigravida	47/73 (64.38%)
Duration of the second stage of labor (min)	85 min
Newborn weight (kg)	2.79 ± 0.43 kg

**Breastfeeding and resting positions VAS**

The lowest VAS score during breastfeeding was in left lateral position (14.51 ± 11.65), and the highest VAS score was in crossed-leg position (74.82 ± 28.33) (p = 0.0001) (Table 2). The lowest VAS score in routine activities was in left lateral position (21.25 ± 13.95), and the highest VAS score was in sitting with leg down position (56.52 ± 21.23) (p = 0.0001) (Table 2).

**Table 2:** VAS During Breastfeeding Positions and Activities VAS, Visual Analog Scale.

Breastfeeding Positions	VAS	p Value
Left lateral	14.5	< 0.0001
Sitting with legs straight on bed	19.14	
Right lateral	29.53	
Reclining position	35.21	
Sitting with legs down	56.62	
Sitting with legs crossed	74.82	
Activities		
Left lateral (OA)	21.25	< 0.0001
Sitting with legs straight (OA)	28.72	
Right lateral (OA)	34.79	
Supine (OA)	35.81	
Walking (OA)	43.67	
Sitting with legs down (OA)	56.52	
Toilet		
Western (severe pain)	5/73 (6.1%)	< 0.0001
Indian (severe pain)	46/73 (69.70%)	

The VAS score increased as birth weight of the baby increased during breastfeeding (p = 0.796) and other activities (p = 0.302). The VAS score increased as duration of second stage increased during different breastfeeding positions (p = 0.92) and other activities (p = 0.047). While using Indian toilet, 46/73 (69.70%) of women had severe pain, whereas only 5/73 (6.1%) had severe pain while using Western toilets (p ≤ 0.0001) (Table 3).

**Table 3:** Effect of Birth Weight and Duration of Second Stage of Labor on VAS VAS, Visual Analog Scale.

	Mean VAS Scores	p Value
Birth weight (kg)	Breastfeeding in crossed leg	0.796
2.5-3	76	
3.1-3.5	76	
3.6-4	95.2	
Birth weight (kg)	Lying down	
2.5-3	44.45	0.302
3.1-3.5	49.93	
3.6-4	50.23	
Duration of second stage of labor (hours)	Crossed leg in breastfeeding	
0.25-0.50	42.96	0.92
0.51-0.75	23.75	
0.76-1.0	31.16	
1.1-1.25	50	
1.26-1.50	27.22	
1.51-1.75	30	
1.76-2.0	60	
2.1-2.25	32.5	
Duration of second stage of labor (hours)	Lying down	
0.25-0.50	75.54	0.047
0.51-0.75	66.25	
0.76-1.0	73.76	
1.1-1.25	71	
1.26-1.50	86.67	
1.51-1.75	67.5	
1.76-2.0	60	
2.1-2.25	65	

## Discussion

The episiotomy is done during second stage of labor to expand the opening of the vagina, which improves maternal and neonatal outcomes. The frequency of episiotomy among primiparous women reported at our center is about 76%-80%.

The most common adverse effect of episiotomy is perineal pain. The reported frequency of perineal pain in a study on the first, 10th, 40th, and 90th day after birth was 96.4%, 63%, 25%, and 12%, respectively [5]. In our study also, almost all patients have the perineal pain related to episiotomy.

The episiotomy pain can result in mother’s inability to take care of the newborn and can lead to insomnia, fatigue, and ignoring education about the infant care especially among primiparous women. Thus it is important to treat perineal pain by pharmacological or non-pharmacological methods. The non-drug methods used commonly are hot and cold compression. Drugs used commonly to relieve pain are acetaminophen, aspirin, ibuprofen, and diclofenac sodium. The drug therapy not only has maternal adverse effects but also can affect the new born as these drugs are excreted through the breast milk [5-7].

In our study, severity of perineal pain during breastfeeding was least when it was done in left lateral position. Similarly, for sleeping or any other routine activity, left lateral position was least painful followed by lying supine position for episiotomy-related perineal pain. Thus, one can advise postnatal females these positions for breastfeeding and kangaroo care. This will be helpful in starting breastfeeding early and better care of newborn. This will have positive impact on maternal health too, as pain can affect mental and physical health of postnatal females. There are no previous studies available regarding the relation of position to episiotomy-related perineal pain.

The severe perineal pain was more common with the usage of Indian-style toilets as compared to Western-style toilets. The majority of Indian homes have Indian-style toilets, thus one can advise them to use toilet seats for better postpartum care. In this study the association of severity of perineal pain to the birth weight of new born and duration of second stage of labor were not found to be significant.

There are few limitations to our study. As it is a single-center study with small sample size, results cannot be generalized. Limiting the use of episiotomy to strict indications has a number of benefits: less posterior perineal trauma, less need for suturing, and fewer complications. Episiotomy does not lead to reduction in most pain measures and severe vaginal or perineal trauma, although it may increase the risk of anterior perineal trauma. The type of suture and the technique of suturing effect on pain severity were also not assessed because in previous studies, subcuticular suturing and Dexon sutures were associated with less perineal pain [6,7]. Also the effect of most commonly assumed posture to healing of episiotomy was not evaluated.

## Conclusions

To conclude, our study shows that on first postnatal day the left lateral position is associated with least perineal pain during breastfeeding and routine activities. So the left lateral position can be advised to the puerperal females for breastfeeding and resting supine as these are the least painful positions. Also the Western-style toilets are more comfortable than Indian-style toilets.
